# Educational intervention for family caregivers of older adults after stroke: pragmatic trial protocol

**DOI:** 10.1590/0034-7167-2024-0234

**Published:** 2025-03-14

**Authors:** Débora Francisco do Canto, Francine Melo da Costa, Amália de Fátima Lucena, Lisiane Manganelli Girardi Paskulin

**Affiliations:** I Universidade Federal do Rio Grande do Sul. Porto Alegre, Rio Grande do Sul, Brazil

**Keywords:** Caregivers, Stroke, Educational Technology, Hospital to Home Transition, Aged, Cuidadores, Accidente Cerebrovascular, Tecnología Educacional, Transición del Hospital al Hogar, Anciano

## Abstract

**Objectives:**

to describe an educational intervention using digital technology for family caregivers of older adult stroke survivors to develop caregiving skills.

**Methods:**

randomized pragmatic trial protocol. Fifty-eight family caregivers will be recruited and randomized into two groups. The intervention will consist of a virtual educational action, in addition to telephone contacts at seven, 30, 60 and 80 days after discharge. The control group will not receive the intervention. Primary outcomes will be caregivers’ ability to care, knowledge and performance. Secondary outcomes will be caregiver burden and older adults’ functional capacity. Outcomes will be assessed at the time of participant recruitment and three months after discharge.

**Results:**

this research aims to provide support that demonstrates the effectiveness of virtual interventions as a means of education and development of care skills.

**Conclusions:**

it is expected that the intervention will contribute to training caregivers and reducing their burden.

## RESUMEN

**Objetivos:**

describir una intervención educativa con tecnología digital para cuidadores familiares de ancianos sobrevivientes de un accidente cerebrovascular para desarrollar habilidades de cuidado.

**Métodos:**

protocolo pragmático de ensayo aleatorio. Se reclutarán y asignarán al azar a 58 cuidadores familiares en dos grupos. La intervención consistirá en una acción educativa virtual, además de contactos telefónicos a los siete, 30, 60 y 80 días tras el alta. El grupo de control no recibirá la intervención. Los resultados primarios serán la capacidad de cuidar, el conocimiento y el desempeño de los cuidadores. La carga del cuidador y la capacidad funcional del anciano serán resultados secundarios. Los resultados se evaluarán en el momento del reclutamiento de los participantes y tres meses después del alta.

**Resultados:**

esta investigación tiene como objetivo ofrecer apoyo que demuestre la efectividad de las intervenciones virtuales como medio de educación y desarrollo de habilidades de cuidado.

**Conclusiones:**

se espera que la intervención contribuya a empoderar al cuidador y reducir su carga.

## INTRODUCTION

The proportion of individuals diagnosed with stroke increases with age, being more prevalent in older adults. In Brazil, in 2022, stroke accounted for 35,982 deaths, 88% of which were in individuals aged 60 or over^(1)^.

After discharge, stroke survivors often return home, where they rely on emotional and physical support from caregivers. In most cases, these family caregivers do not have the knowledge, training, or resources to provide the necessary care^(2)^. In Brazil, there are currently no structured support programs for family caregivers, and there is a gap in national health policies aimed at home care^(3)^.

In southern Brazil, the Nursing Home Care Intervention Post Stroke (SHARE) study offered an educational intervention to family caregivers of older adults after stroke through home visits within one month after hospital discharge. The intervention consisted of preparing caregivers to perform activities of daily living (ADLs) for older adults, providing emotional support, and providing guidance on using healthcare services. The results showed a low impact of the intervention on the outcomes of interest. The study did not assess caregivers’ ability to provide care, so the authors suggested that research be conducted to assess caregivers’ ability^(3)^.

In the international scenario, a cross-sectional study conducted in Malaysia aimed to determine the level of knowledge among caregivers and their level of confidence in caring for patients after a stroke. The findings reinforce the need for training programs for caregivers and policies that ensure their implementation in healthcare services^(2)^.

Recently, due to the COVID-19 pandemic, many healthcare and research activities have been suspended or altered. During this period, greater vulnerability was identified in older adults as the age group with the highest risk of death, especially those with comorbidities, such as stroke. To meet this demand, healthcare and research strategies in virtual environments have been intensified^(4)^.

In the local context, there is no evidence that an educational intervention based on skills development can improve caregivers’ ability, knowledge, and performance to provide care to older adults who have survived stroke. Thus, the present study considers the hypotheses that: (1) the educational intervention with digital technology, carried out by nurses for family caregivers of older adults with stroke sequelae, improves caregivers’ ability to care, performance, and knowledge in the intervention group (IG) when compared to caregivers in the control group (CG); (2) the intervention offered reduces caregiver burden; (3) older adults in the IG will present better functional capacity when compared to the CG.

## OBJECTIVES

To describe the protocol of an educational intervention using digital technology for family caregivers of older adults who are stroke survivors to develop home care skills.

## METHODS

This is a randomized, blinded, pragmatic trial (PT) with three-month follow-up after hospital discharge, called “Digital educational intervention for family caregivers of older adults after stroke (e-SHARE)”. All stages are in accordance with CONsolidate Standards Of Reporting Trials (CONSORT) extension for PT recommendations. The protocol is based on the Standard Protocol Items: Recommendations for Interventional Trials (SPIRIT 2013 Statement).

### Study location

The study is being developed in the emergency service, in the clinical inpatient units and in the Special Care Unit - Stroke of a university hospital, a reference for the care of stroke patients in the city of Porto Alegre, Rio Grande do Sul, Brazil.

### Eligibility criteria

Study participants are family caregivers of older adults selected at hospital admission who play the role of primary care provider. Family caregivers of older adults will be excluded from the study if: (a) they live in a long-term care facility; (b) they do not have access to the internet and a device to access it, or are not able to use them, as verified through a digital aptitude checklist developed for the study; (c) they do not have a telephone line for contact; (d) they are accompanying older adults who died during the participant recruitment phase.

### Intervention

The educational intervention will be carried out by two nurses, through an online course in the format of Massive Open and Online Courses (MOOCs). MOOCs are teaching tools that provide flexible hours, quality material and low cost for implementation^(5)^.

The MOOC for family caregivers was developed digitally and made available on the Moodle Platform, since this platform provides access statistics. During the study period, access will be restricted to IG members. MOOC development is based on the Manual for Caregivers of Older Adults with Stroke^(6)^. The MOOC includes 12 modules, which can be accessed as many times as necessary by caregivers, in random order, according to older adults’ and caregivers’ care needs. Telephone contacts will also be made with IG participants seven days, 30 days, 60 days and 80 days after hospital discharge and a hotline will be made available for contact.

### Results

The intervention aims to equip family caregivers to assist older adults in ADLs after hospital discharge.

### Participant timeline

**Figure 1:**
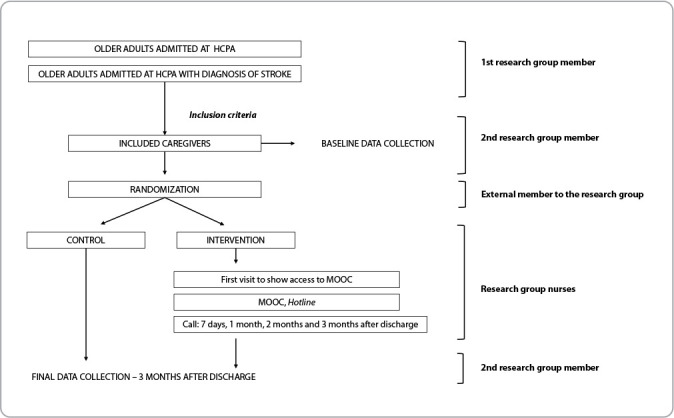
Graphical representation of study logistics diagram and operationalization of data collection, Porto Alegre, Rio Grande do Sul, Brazil, 2024

Figure 1 presents the study logistics diagram.

The study will begin with the identification of patients aged 60 or older who have a medical diagnosis of stroke during their current hospitalization. The older adults selected will receive a visit from the researchers to verify the existence of a family caregiver and to assess whether the caregiver and the older adult meet the inclusion criteria. Baseline data will be collected for those who agree to participate. After allocation, those included in the IG will receive an instructional visit from the intervention nurses close to discharge. The purpose of the visit is to provide detailed guidance on the intervention.

Caregivers will be contacted by telephone three months after discharge to conduct the final assessment. Data collection will be done via online conference. The three-month interval meets Brazilian reproducibility study of the Scale of Informal Caregiver Capabilities for Older Adults Dependent on Stroke (ECCIID-AVC) recommendations, which will be used in the study^(7)^.

### Sample size

Sample size was calculated, considering a power of 80%, a significance level of 5% and a Cohen’s effect size of 0.8, adding 10% for possible losses and refusals, totaling 58 participants, divided equally into IG and CG.

### Recruitment

Participants will be recruited through daily consultation of the hospital’s computerized system.

### Allocation

After the instruments are applied, participants will be randomized to either the CG or IG group. To do this, a list generated by the website randomizer.org will be used. A professional external to the research group will be responsible for the list.

### Blinding

Researchers who will perform the initial and final assessment of participants will be blind to allocation.

### Data collection

Data collection will be performed at baseline and three months later. A structured questionnaire was developed with sociodemographic and clinical information from family caregivers and older adults. All information regarding older adults will be provided by caregivers. Instruments that assess caregivers’ ability to care, ECCIID-AVC, their knowledge and performance in caring for older adults (Nursing Outcomes Classification (NOC)) as well as instruments that assess the burden of these caregivers (Caregiver Bunder Scale (CBS)) and the measure of functional independence of older adults (Functional Independence Measure (FIM)) will also be applied.

Regarding the instruments used in the study, the ECCIID-AVC assesses the different capabilities that family caregivers have or need to improve when providing care to dependent older adults after a stroke. In the reproducibility study of the Brazilian version, the ECCIID-AVC showed satisfactory test-retest reliability (Intraclass Correlation Coefficient = 0.94; 95% Confidence Interval = 0.91-0.96) and excellent internal consistency reliability (Cronbach’s alpha = 0.914)^(7)^.

The NOC is a standardized taxonomy for nursing-sensitive outcomes intended for clinical practice and with the objective of verifying changes in the condition of a patient, family or community after an applied nursing intervention^(8)^. For the present study, the outcomes “Knowledge: Stroke Management” and “Caregiver Performance: Direct Care” were selected. A group of ten experts with knowledge and experience in using this classification assisted the researchers in selecting the indicators for these two outcomes. For each selected indicator, a conceptual definition and an operational definition were constructed.

Family caregivers’ burden will be assessed using the CBS. This scale was adapted and validated for the Brazilian context by Medeiros *et al*.^(9)^ with a population of caregivers of patients with rheumatic diseases. The intraand inter-observer reproducibility coefficients were 0.87 and 0.92, respectively, and the Portuguese version proved to be a valid instrument^(9)^.

Older adults’ functional capacity will be assessed using the FIM. The scale was translated into Brazilian Portuguese in 2000 and underwent validity for the Brazilian context in 2004. The Brazilian version of the scale has good cultural equivalence and good reproducibility^(10)^.

### Statistical methods

For data analysis, the intention-to-treat technique will be used. The difference between baseline and final scores will be assessed by paired Student’s t-test, with a 95% Confidence Interval. A p<0.05 value will be considered significant. For simultaneous intraand intergroup comparisons, the Generalized Estimating Equations model with Bonferroni adjustment will be used.

### Data monitoring

All researchers involved in the study were properly trained and tested to assess agreement in the application of the instruments performed.

### Damages

The investigation process may cause fatigue and some discomfort to participants, in addition to possibly increasing mobile internet data consumption.

### Ethical approval in research

The project was approved by the Research Ethics Committee, under *Certificado de Apresentação para Apreciação Ética* (CAAE, Certificate of Presentation for Ethical Consideration), and its protocol is registered at clinicaltrial.gov.

### Consent

An Informed Consent Form (ICF) will be applied to family caregivers, and the voluntary nature of participation as well as anonymity will be ensured.

### Confidentiality

To access the information in medical records, legal requirements must be met and a term of commitment for use must be signed.

### Declaration of interests

The authors report no conflicts of interest.

### Data access

Study data will be available for access.

### Disclosure policy

The results will be disclosed to the services in which the research will be carried out, to participants and to the scientific community.

## RESULTS

This research aims to provide support that demonstrates the effectiveness of virtual interventions as a means of education and development of care skills aimed at family caregivers of older adults who have suffered a stroke. This is a pioneering study in the country that values nurses’ educational role.

## DISCUSSION

Stroke is more common in older adults, and its consequences include motor, sensory and cognitive function disorders that reduce the ability to perform ADLs. After discharge, stroke survivors usually return home, where they can count on the support of caregivers.

Nurses play a fundamental role in educating stroke survivors and their family caregivers during hospitalization and in preparing for discharge, through guidance on the pathology, emotional support for caregivers and older adults, use of services available in the health network, performance of care activities and reduction of caregiver burden. Educational materials can facilitate this process^(6)^, and the educational manual used as the basis for the intervention proposed in this study is the first developed in the Brazilian context for family caregivers of older adults after stroke.

Interventions using digital technology have been described in the literature since 2010 through the use of telephones, video calls and, more recently, other technologies such as apps. The restrictions imposed by the pandemic have made the use of these resources indispensable, representing new forms of continuity of care. In developing countries, such as Brazil, these interventions still need to be further tested. In this scenario, MOOCs represent a positive educational alternative that deserves to be further explored in the health area^(5)^.

The use of PT is still in its infancy in nursing research, and this method is considered robust and adequate to assess the effect of the proposed intervention. The ECCIID-AVC, recently adapted and validated for use in Brazil, will be used for the first time in this study, assessing family caregivers’ ability to care. The choice of NOC to assess the outcomes of caregiver knowledge and performance also stands out as an innovation in this research, since there are currently no similar studies. The digital educational intervention, developed by nurses especially for this study, will be made available at the end of the research as a complementary tool for the education of patients and caregivers.

### Study limitations

Limitations of this study may include the fact that participants were recruited from a referral institution for stroke care that offers specialized care through a multidisciplinary team. Other possible limitations may include loss to follow-up due to death of stroke survivors, admission to long-term care facilities, or change of caregivers.

### Contributions to health, nursing or public policy

This research presents advances in nursing knowledge by promoting educational alternatives aimed at family caregivers of older adults who have suffered a stroke in the Brazilian context, constituting a technology for nursing assistance in transition of care.

## CONCLUSIONS

This study presented the protocol of a randomized PT aimed at family caregivers of older adult stroke survivors that aims to explore alternatives for transition of care from hospital to home with the support of digital technology.
