# Supplementary material for: Educational intervention for family caregivers of older adults after stroke: pragmatic trial protocol
Source: Rev Bras Enferm. 2025 Mar 14;78(1):e20240234. doi: 10.1590/0034-7167-2024-0234 (PMC11913040; doi:10.1590/0034-7167-2024-0234)
Supplement: Translation

# Translation: Intervenção educativa para cuidadores familiares de idosos após acidente vascular cerebral: protocolo de ensaio pragmático

**DOI:** 10.1590/0034-7167-2024-0234pt

**Authors:** Débora Francisco do Canto, Francine Melo da Costa, Amália de Fátima Lucena, Lisiane Manganelli Girardi Paskulin

**Affiliations:** I Universidade Federal do Rio Grande do Sul. Porto Alegre, Rio Grande do Sul, Brasil

**Keywords:** Cuidadores, Acidente Vascular Cerebral, Tecnologia Educacional, Transição do Hospital para o Domicílio, Idoso

## Abstract

**Objetivos:**

descrever uma intervenção educativa com tecnologia digital para cuidadores familiares de idosos sobreviventes de acidente vascular cerebral para o desenvolvimento de habilidades de cuidado.

**Métodos:**

protocolo de ensaio pragmático randomizado. Serão recrutados 58 cuidadores familiares e randomizados em dois grupos. A intervenção consistirá em uma ação educativa virtual, além de contatos telefônicos em sete, 30, 60 e 80 dias após a alta. O grupo controle não receberá a intervenção. Serão desfechos primários a capacidade de cuidar, o conhecimento e o desempenho dos cuidadores. Serão desfechos secundários a sobrecarga do cuidador e a capacidade funcional do idoso. Os desfechos serão avaliados na captação dos participantes e três meses após a alta.

**Resultados:**

esta pesquisa pretende oferecer subsídios que demonstrem a efetividade de intervenções virtuais como meio de educação e desenvolvimento de habilidades de cuidado.

**Conclusões:**

espera-se que a intervenção contribua para a capacitação do cuidador e redução da sua sobrecarga.

## INTRODUÇÃO

A proporção de indivíduos com diagnóstico de acidente vascular cerebral (AVC) aumenta conforme o avanço da idade, sendo mais prevalente em idosos. No Brasil, em 2022, o AVC foi responsável por 35.982 mortes, sendo 88% em indivíduos com 60 anos ou mais^(1)^.

Após a alta, os sobreviventes de AVC geralmente retornam para o domicílio, onde contam com apoio emocional e físico dos cuidadores. Na maioria dos casos, esses cuidadores familiares não têm conhecimento, treinamento ou recursos para prestar o cuidado necessário^(2)^. No Brasil, atualmente, não existem programas estruturados de suporte aos cuidadores familiares, havendo uma lacuna no âmbito das políticas nacionais de saúde voltadas ao atendimento domiciliar^(3)^.

No sul do Brasil, o estudo *Nursing Home Care Intervention Post Stroke* (SHARE) ofereceu intervenção educativa aos cuidadores familiares de idosos após AVC por meio de visitas domiciliares no período de um mês após a alta hospitalar. A intervenção consistiu no preparo dos cuidadores para a realização das atividades de vida diária (AVDs) do idoso, no suporte emocional e em orientações para a utilização dos serviços de saúde. Os resultados demonstraram baixo impacto da intervenção nos desfechos de interesse. O estudo não avaliou a habilidade dos cuidadores em cuidar, de forma que os autores sugeriram a realização de pesquisas que avaliassem a capacidade do cuidador^(3)^.

No cenário internacional, estudo transversal, realizado na Malásia, objetivou determinar o nível de conhecimento entre os cuidadores e seu nível de confiança no cuidado ao paciente após um AVC. Os achados reforçam a necessidade de programas de treinamento para os cuidadores e políticas que garantam a sua implementação nos serviços de saúde^(2)^.

Recentemente, em função da pandemia de COVID-19, muitas atividades assistenciais e de pesquisa foram suspensas ou alteradas. Neste período, identificou-se maior vulnerabilidade nos idosos enquanto grupo etário com mais risco de morte, especialmente aqueles com comorbidades, como o AVC. Para suprir essa demanda, estratégias assistenciais e de pesquisa em ambientes virtuais têm se intensificado^(4)^.

No contexto local, não há evidências se uma intervenção educativa baseada no desenvolvimento de habilidades pode melhorar a capacidade, o conhecimento e o desempenho dos cuidadores para realizar a atenção ao idoso sobrevivente de AVC. Assim, o presente estudo considera as hipóteses de que: (1) a intervenção educativa com tecnologia digital, realizada por enfermeiros para cuidadores familiares de idosos com sequela de AVC, melhora a capacidade de cuidar, o desempenho e o conhecimento dos cuidadores do grupo intervenção (GI), quando comparados aos cuidadores do grupo controle (GC); (2) a intervenção oferecida reduz a sobrecarga do cuidador; (3) os idosos do GI irão apresentar melhor capacidade funcional quando comparados ao GC.

## OBJETIVOS

Descrever o protocolo de uma intervenção educativa com tecnologia digital para cuidadores familiares de idosos sobreviventes de AVC para o desenvolvimento de habilidades de cuidado no domicílio.

## MÉTODOS

Trata-se de ensaio pragmático (EP) randomizado, cego e com acompanhamento por três meses após a alta hospitalar, denominado “Intervenção educativa digital para cuidadores familiares de idosos após acidente vascular cerebral (e-SHARE)”. Todas as etapas estão de acordo com as recomendações da extensão do *CONsolidate Standards Of Reporting Trials* (CONSORT) para EPs. O protocolo está baseado no *Standard Protocol Items: Recommendations for Interventional Trials* (*SPIRIT 2013 Statement*).

### Local do estudo

O estudo está sendo desenvolvido no serviço de emergência, nas unidades de internação clínica e na Unidade de Cuidado Especiais - AVC de um hospital universitário, referência para o atendimento de pacientes com AVC na cidade de Porto Alegre, Rio Grande do Sul, Brasil.

### Critérios de elegibilidade

Os participantes do estudo são os cuidadores familiares de idosos selecionados no momento da internação hospitalar que exercem o papel de principal provedor de cuidados. Serão excluídos do estudo os cuidadores familiares de idosos que: (a) residirem em instituição de longa permanência; (b) não tiverem acesso à internet e aparelho para acesso, ou que não estiverem aptos a utilizá-los, verificado através de *checklist* de aptidão digital elaborado para o estudo; (c) não tiverem linha telefônica para contato; (d) estiverem acompanhando idosos que forem a óbito na fase de captação dos participantes.

### Intervenção

A intervenção educativa será realizada por duas enfermeiras, por meio de um curso *online* no formato de Cursos Massivos Abertos e *Online* (MOOCs, sigla em inglês para *Massive Open and Online Courses*). MOOCs são ferramentas de ensino que proporcionam flexibilidade de horário, material de qualidade e baixo custo para realização^(5)^.

O MOOC para os cuidadores familiares foi desenvolvido digitalmente e disponibilizado na Plataforma Moodle, uma vez que esta fornece estatísticas de acesso. Durante o período do estudo, ele será de acesso restrito aos membros do GI. O desenvolvimento do MOOC tem como base o Manual para Cuidadores de Idosos com AVC^(6)^. O MOOC contempla 12 módulos, que poderão ser acessados quantas vezes forem necessárias pelos cuidadores, em ordem aleatória, de acordo com as necessidades de cuidado do idoso e do próprio cuidador. Também serão realizados contatos telefônicos com os participantes do GI em sete dias, 30 dias, 60 dias e 80 dias após a alta hospitalar e disponibilizada uma *hotline* para contato.

### Resultados

A intervenção visa instrumentalizar o cuidador familiar para assistir o idoso nas AVDs após a alta hospitalar.

### Linha do tempo do participante

O diagrama da logística do estudo está apresentado na Figura 1.

**Figura 1:** Representação gráfica do diagrama da logística do estudo e operacionalização da coleta de dados, Porto Alegre, Rio Grande do Sul, Brasil, 2024

A pesquisa iniciará com a identificação dos pacientes com 60 anos ou mais e diagnóstico médico de AVC na internação atual. Os idosos captados receberão uma visita dos pesquisadores para verificar a existência de cuidador familiar e para avaliar se ele e o idoso atendem aos critérios de inclusão. Para os que aceitarem participar, serão coletados os dados basais. Os incluídos no GI, após a alocação, receberão uma visita de instrução, pelas enfermeiras intervencionistas, próxima à alta. O objetivo da visita é fornecer orientações detalhadas sobre a intervenção.

O cuidador será contatado via telefone três meses após alta para a realização da avaliação final. A coleta se dará via conferência *online*. O intervalo de três meses atende ao recomendado pelo estudo de reprodutibilidade brasileira da Escala de Capacidades do Cuidador Informal de Idosos Dependentes por AVC (ECCIID-AVC), que será utilizada no estudo^(7)^.

### Tamanho da amostra

Foi calculado o tamanho da amostra, considerando-se poder de 80%, nível de significância de 5% e tamanho de efeito de Cohen de 0.8, acrescentando-se 10% para possíveis perdas e recusas, totalizando 58 participantes, divididos igualmente em GI e GC.

### Recrutamento

Os participantes serão recrutados através de consulta diária ao sistema informatizado do hospital.

### Alocação

Após a aplicação dos instrumentos, os participantes serão randomizados para grupo GC ou GI. Para tal, será utilizada uma lista gerada pela página eletrônica *randomizer.org*. Um profissional externo ao grupo de pesquisa ficará responsável pela lista.

### Cegamento

Os pesquisadores que realizarão a avaliação inicial e final dos participantes serão cegos para a alocação.

### Coleta de dados

A coleta de dados será realizada na avaliação basal e três meses após. Foi desenvolvido um questionário estruturado com informações sociodemográficas e clínicas dos cuidadores familiares e dos idosos. Todas as informações referentes aos idosos serão fornecidas pelos cuidadores. Também serão aplicados instrumentos que avaliem a capacidade de cuidar dos cuidadores, ECCIID-AVC, seu conhecimento e desempenho no cuidado ao idoso (*Nursing Outcomes Classification* (NOC)), além de instrumentos que avaliem a sobrecarga desses cuidadores (*Caregiver Bunder Scale* (CBS)) e a medida de independência funcional dos idosos (Medida de Independência Funcional (MIF)).

Quanto aos instrumentos utilizados no estudo, o ECCIID-AVC avalia as diferentes capacidades que os cuidadores familiares têm ou precisam melhorar na prestação de cuidados a idosos dependentes após um AVC. No estudo de reprodutibilidade da versão brasileira, a ECCIID-AVC apresentou confiabilidade teste-reteste satisfatória (Coeficiente de Correlação Intraclasse = 0,94; Intervalo de Confiança de 95% = 0,91-0,96) e uma excelente confiabilidade de consistência interna (alfa de Cronbach = 0,914)^(7)^.

A NOC é uma taxonomia padronizada para os resultados sensíveis à enfermagem destinada à prática clínica e com o objetivo de verificar alterações na condição do paciente, família ou comunidade após uma intervenção de enfermagem aplicada^(8)^. Para o presente estudo, foram selecionados os resultados “Conhecimento: Controle do Acidente Vascular Encefálico” e “Desempenho do Cuidador: Cuidados Diretos”. Um grupo de dez especialistas com conhecimento e experiência na utilização desta classificação auxiliou os pesquisadores na seleção dos indicadores desses dois resultados. Para cada indicador selecionado, uma definição conceitual e uma definição operacional foi construída.

A sobrecarga do cuidador familiar será verificada através da CBS. Esta escala foi adaptada e validada ao contexto brasileiro por Medeiros *et al*.^(9)^ com uma população de cuidadores de pacientes com doenças reumáticas. Os coeficientes de reprodutibilidade intra e inter observador foram 0,87 e 0,92, respectivamente, e a versão em português se mostrou um instrumento válido^(9)^.

A capacidade funcional dos idosos será avaliada com a MIF. A escala foi traduzida para o português brasileiro em 2000, e passou por processo de validação para o contexto brasileiro em 2004. A versão brasileira da escala possui boa equivalência cultural e boa reprodutibilidade^(10)^.

### Métodos estatísticos

Para a análise dos dados, será utilizada a técnica por intenção de tratar. A diferença dos escores basais e finais será avaliada pelo teste t de *Student* pareado, com Intervalo de Confiança de 95%. Será considerado como valor significativo p<0,05. Para as comparações intra e intergrupos simultaneamente, o modelo de Equações de Estimativas Generalizadas com ajuste por Bonferroni será utilizado.

### Monitoramento de dados

Todos os pesquisadores envolvidos no estudo foram devidamente treinados e testes para a avaliação da concordância na aplicação dos instrumentos realizados.

### Danos

O processo de investigação pode gerar cansaço e algum desconforto aos participantes, além de maior consumo de dados móveis de internet, possivelmente.

### Aprovação ética em pesquisa

O projeto foi aprovado pelo Comitê de Ética em Pesquisa, sob Certificado de Apresentação para Apreciação Ética (CAAE), e seu protocolo está cadastrado no *clinicaltrial.gov*.

### Consentimento

Será aplicado um Termo de Consentimento Livre e Esclarecido (TCLE) aos cuidadores familiares e assegurado o caráter voluntário de participação, bem como o anonimato.

### Confidencialidade

Para acesso às informações no prontuário, serão cumpridos os requisitos legais e assinado um termo de compromisso para utilização.

### Declaração de interesses

Os autores informam não possuir conflitos de interesse.

### Acesso aos dados

Os dados do estudo ficarão disponíveis para acesso.

### Política de divulgação

Os resultados serão divulgados para os serviços nos quais a pesquisa será realizada, para os participantes e para a comunidade científica.

## RESULTADOS

A presente investigação pretende oferecer subsídios que demonstrem a efetividade de intervenções virtuais como meio de educação e desenvolvimento de habilidades de cuidado voltada a cuidadores familiares de idosos que sofreram AVC. Trata-se de estudo pioneiro no país que valoriza o papel educativo dos enfermeiros.

## DISCUSSÃO

O AVC é mais incidente em idosos, e suas sequelas incluem distúrbios das funções motoras, sensoriais e cognitivas que reduzem a capacidade de desempenhar as AVDs. Após a alta, os sobreviventes de AVC geralmente retornam para o domicílio, onde contam com o apoio dos cuidadores.

O enfermeiro desempenha um papel fundamental na educação dos sobreviventes ao AVC e seus cuidadores familiares durante a hospitalização e no preparo para a alta, através de orientações sobre a patologia, suporte emocional ao cuidador e ao idoso, utilização dos serviços disponíveis na rede de saúde, realização das atividades de cuidado e redução da sobrecarga do cuidador. Os materiais educativos podem ser facilitadores neste processo^(6)^, sendo o manual educativo utilizado como base para a intervenção proposta neste estudo o primeiro desenvolvido, no contexto brasileiro, para cuidadores familiares de idosos após AVC.

As intervenções com tecnologia digital são descritas na literatura desde 2010 através do uso do telefone, das chamadas de vídeo e, mais recentemente, de outras tecnologias, como os aplicativos. As restrições impostas pela pandemia tornaram o uso desses recursos indispensável, representando novas modalidades de continuidade de cuidado. Nos países em desenvolvimento, como o Brasil, essas intervenções ainda precisam ser mais bem testadas. Neste cenário, os MOOCs representam uma alternativa educacional positiva que merece ser mais explorada na área da saúde^(5)^.

EPs ainda são incipientes na pesquisa em enfermagem, e considera-se que este método é robusto e adequado para avaliar o efeito da intervenção proposta. A ECCIID-AVC, recentemente adaptada e validada para uso no Brasil, será utilizada pela primeira vez neste estudo, avaliando a capacidade de cuidar dos cuidadores familiares. A escolha da NOC para avaliação dos desfechos conhecimento e desempenho do cuidador também se destaca como inovação nesta pesquisa, visto que não há estudos semelhantes atualmente. A intervenção educativa digital, construída por enfermeiras especialmente para este estudo, será disponibilizada ao término da pesquisa como uma ferramenta complementar para a educação de pacientes e cuidadores.

### Limitações do estudo

Poderão constituir limitações deste trabalho o fato de os participantes serem captados em uma instituição de referência para o atendimento de AVC que oferece assistência especializada por meio de equipe multidisciplinar. Outras possíveis limitações podem incluir a perda de acompanhamento devido à morte dos sobreviventes do AVC, internação em instituições de longa permanência ou troca de cuidadores.

### Contribuições para as áreas da saúde, enfermagem ou políticas públicas

Esta pesquisa apresenta avanços no conhecimento de enfermagem, ao promover alternativas educacionais voltadas a cuidadores familiares de idosos que sofreram AVC no contexto brasileiro, constituindo uma tecnologia à assistência de enfermagem na transição do cuidado.

## CONCLUSÕES

Este estudo apresentou o protocolo de um EP randomizado voltado a cuidadores familiares de idosos sobreviventes de AVC que visa explorar alternativas para a transição do cuidado do hospital para o domicílio com suporte de tecnologia digital.
